# Research on the impact of tourism cooperation on urban public health

**DOI:** 10.3389/fpubh.2025.1556789

**Published:** 2025-03-05

**Authors:** Liang Zhao, Guolei Zhou, Chen Li

**Affiliations:** ^1^School of Tourism, Hubei University, Wuhan, China; ^2^School of Business, Hubei University, Wuhan, China; ^3^School of Management, Shanghai University of Engineering Science, Shanghai, China

**Keywords:** tourism cooperation, urban public health, tourism health, cities, China

## Abstract

**Introduction:**

Tourism cooperation is increasingly recognized as a key driver of regional economic development, playing a crucial role in facilitating internal circulation and enhancing urban public health. This study examines the relationship between tourism cooperation and urban public health development, with a focus on China.

**Methods:**

Using data from 284 Chinese cities over the period 2011-2022, this research measures both the degree of tourism cooperation and the level of urban public health development. Empirical analysis is conducted to assess the impact of tourism cooperation on urban public health outcomes. The study employs robustness tests to ensure the validity of its findings.

**Results:**

The findings indicate that tourism cooperation significantly promotes urban public health development. After conducting robustness tests, this result remains consistent. Furthermore, heterogeneity analysis reveals that the “core hinterland” type of tourism cooperation model has a stronger impact on urban public health development compared to the adjacent city type. Among different city cooperation models, tourism cooperation between central cities has the greatest empowering effect, followed by cooperation between central and non-central cities, while cooperation between non-central cities has the smallest effect.

**Discussion:**

The research suggests that tourism cooperation can effectively address regional economic disparities and the imbalanced development of urban public health in China. The findings have important implications for policy-making, particularly in promoting sustainable urban health development and narrowing regional gaps in economic and health outcomes.

## 1 Introduction

Tourism Cooperation refers to the joint promotion of sustainable development in the tourism industry among different countries, regions, or organizations through resource sharing, information exchange, policy coordination, and other means. This type of collaboration typically involves multiple stakeholders, including government, businesses, non-governmental organizations (NGOs), and local communities. The core goal of tourism cooperation is to achieve mutual benefit and win-win in the tourism industry, enhance the competitiveness of tourism destinations, and protect natural and cultural resources. Regional tourism cooperation refers to the development of cross-border tourism routes, sharing of tourism resources, and coordination of tourism policies among neighboring or adjacent countries or regions to enhance the overall tourism attractiveness of the region. For example, the “Danube Tourist Area” cooperation project in Europe. Through regional tourism cooperation, tourist destinations can attract more tourists, increase tourism revenue, and promote local economic development. Different regions or countries can achieve complementary tourism resources through cooperation, such as the combination of natural landscapes and cultural resources. At the same time, tourism cooperation helps to coordinate the relationship between environmental protection and tourism development, avoiding the destruction of natural and cultural resources by overdevelopment (1–4).

Health is a fundamental need for human survival and development, and an important indicator for measuring the progress of social civilization and the overall level of social development. Tourism is a catalyst for promoting human physical and mental health, not only because the essence of tourism is to make people healthy and happy, but also because the tourism health industry can play an important role in promoting human health. The achievement of national health goals requires starting from various aspects such as daily life, services, environment, policies, technological support, and industrial development, to jointly support and promote the development of the national health cause (5–9). The Chinese government has proposed to focus on the development of the health industry and provide comprehensive and full cycle service guarantees and industrial support to promote people’s health.

The tourism industry is a modern service industry aimed at bringing physical and mental health to people and promoting their comprehensive development. It should also play a key role in promoting the development of national health. On the one hand, the essence of tourism is to bring health and happiness to people. The purpose of people traveling is to gain spiritual pleasure, relieve physical fatigue, and achieve physical and mental adjustment while enjoying natural scenery and cultural influence (10–16). A pleasant trip or vacation not only allows people to gain knowledge, broaden their horizons, and cultivate their emotions, but also brings about physical adjustment and promotes physical health through spiritual pleasure. Tourism is actually the best way to promote physical and mental health. This requires us, as managers of the tourism industry and providers of tourism services, to stand at the height of promoting human health, providing high-quality and high-quality industries and services to people, and taking the promotion of human physical and mental health as an indispensable and important responsibility. At the same time, tourism operators should also promote tourism as an important part of “healthy and civilized lifestyle,” and take advocating a healthy and civilized lifestyle as an important social responsibility of tourism operators, guiding the people to promote physical and mental health through tourism (17–20).

On the other hand, the tourism health industry formed by “tourism health” can provide people with the most direct health services and industry support. Vigorously developing new formats of health services, actively promoting the integration and development of the health and older adult care industry with other related industries and fields, and giving birth to new industries, formats, and models of health services; Actively develop the fitness and leisure sports industry, create a comprehensive fitness and leisure service system, actively cultivate fashionable leisure sports projects with consumption leading characteristics such as ice and snow, mountains, water, motorcycle, aviation, extreme sports, and equestrian sports, and create fitness and leisure demonstration zones and fitness and leisure industry belts with regional characteristics. The integration and development of the above-mentioned fields with tourism will directly give rise to the emergence and development of the “tourism health industry” that serves people’s health.

In fact, as society develops and people’s economic income increases, their attention to their physical and mental health becomes higher. Moreover, with the advancement of human consumption and health concepts, this attention has shifted from post illness treatment to health care and prevention, that is, through regular and planned vacations, health care, sub-health rehabilitation and other means to improve their physical and mental health. These health care consumption demands, which are carried out through tourism and vacation, directly promote the “tourism oriented” development of the health industry (21–27). To promote the construction of a “Healthy China” is to take improving the health level of the people as the core, reform and innovation as the driving force, and the development of the health industry as the focus, providing basic guarantees for the development of the national health cause. We need to fully leverage the social influence of tourism and the integration and driving force of the tourism industry, innovate and develop products and formats of the tourism health industry, promote the integrated development of “tourism health,” build a tourism health industry system with Chinese characteristics, meet the multi-level and diversified health service needs of the general public, and make greater contributions to the comprehensive construction of a moderately prosperous society.

The impact of tourism cooperation on urban public health level has a positive effect, firstly, it can enhance public health awareness. Tourism cooperation often accompanies the demand for higher public health standards in tourist destinations, which prompts cities to strengthen public health management and services, and enhance the public health awareness of residents and tourists. Through tourism cooperation, cities can draw on and learn from advanced public health management experiences and technologies from other regions, further enhancing the local public health level (19, 25, 28–30). The second is to promote the construction of medical facilities. Tourism cooperation may lead to the construction and improvement of medical facilities to meet the medical needs of tourists and residents. For example, establishing medical institutions, improving medical treatment capabilities, and perfecting emergency systems. The construction of medical institutions and facilities not only improves the level of medical services in cities, but also enhances their ability to respond to sudden public health emergencies. The third is to improve the urban environment. Tourism cooperation usually requires cities to improve and enhance their tourism environment, including urban greening, beautification, purification, and other aspects. These measures help improve the urban environment and enhance the quality of life for residents and tourists. A good urban environment helps to reduce the occurrence and spread of diseases, thereby improving the public health level of the city. The fourth is to promote the development of the health industry. Tourism cooperation can promote the development of the health industry, such as characteristic health tourism projects such as hot spring therapy and forest health preservation. These projects not only enrich the tourism industry, but also promote the health of urban residents and tourists. The development of the health industry can also drive the extension and upgrading of related industrial chains, injecting new vitality into the urban economy.

Meanwhile, tourism cooperation also has a negative impact on the public health level of cities. On the one hand, population mobility poses public health risks. Tourism cooperation will increase the population mobility of cities, including the flow of tourists and professionals. This increases the risk of the spread of infectious diseases, such as influenza, COVID-19 and other respiratory infectious diseases. Population mobility may also bring challenges to food safety, drinking water safety, and other aspects, requiring cities to strengthen supervision and prevention measures. On the other hand, it leads to uneven distribution of resources (31–34). Tourism cooperation may lead to a tilt of urban resources toward the tourism industry, resulting in uneven distribution of resources in other public service areas. Medical resources may be tight due to the peak tourism season, affecting residents’ medical needs. Therefore, cities need to balance the development of the tourism industry with other public service sectors in tourism cooperation, ensuring the rational allocation and utilization of resources.

Under the basic national conditions of imbalanced development, tourism cooperation, as a new concept of economic development that relies on synergy and system theory to promote the integration and operation of regional economic subsystems, provides opportunities to solve the drawbacks of imbalanced development, unleash domestic demand potential, and promote smooth internal circulation. In recent years, China has gradually explored the application of the concept of tourism cooperation to build a regional economic pattern with complementary advantages and coordinated linkage. It has formulated major regional development strategies such as the coordinated development of Beijing Tianjin Hebei, the development of the Yangtze River Economic Belt, and the Guangdong Hong Kong Macao Greater Bay Area, which have to some extent alleviated the dilemma of imbalanced development. In theory, tourism cooperation can promote factor flow, complementary advantages, and industrial linkage between regions through policy guidance and market mechanisms, thereby solving practical difficulties such as factor mismatch, market segmentation, and industrial homogenization (35, 36). In recent years, studies have examined the impact of tourism cooperation on high-quality economic development and considered the positive significance of tourism cooperation from multiple perspectives. Unfortunately, there is currently no systematic theoretical and empirical research on how tourism cooperation affects the development of urban public health, which may hinder a comprehensive understanding of the intrinsic relationship between tourism cooperation and urban public health development.

The existing research has the following shortcomings: firstly, there is currently a lack of attention paid to the new requirements of regional coordinated development for urban public health development, and there are even fewer studies exploring effective tools to promote urban public health development from this logical starting point. There is still a blank in research on the impact of tourism cooperation on urban public health development. Secondly, although relevant studies have revealed the potential optimization or multiplier effects of tourism cooperation on the constituent elements of urban public health, there is a relative scarcity of research that systematically examines the theoretical mechanisms between the two and further investigates their impact effects. In response to the above shortcomings, this article intends to study the relationship between tourism cooperation, smooth internal circulation, and urban public health. The possible contributions mainly lie in three aspects: firstly, it expands the research on the impact of tourism cooperation on the development of urban public health. By analyzing the cultivation logic and configuration logic of tourism cooperation on the constituent elements of urban public health, as well as the construction logic of the supporting system for urban public health development, a triple nested empowerment theory of tourism cooperation on the development of urban public health is formed. Through empirical testing of the impact effect of tourism cooperation on urban public health development, it provides theoretical basis and empirical reference for solving the regional economic imbalance and insufficiency dilemma faced by China’s urban public health development. Secondly, the heterogeneous characteristics of the impact of tourism cooperation on urban public health development were examined from two dimensions: differences in cooperation modes and cooperation subjects, and differences in external guarantee conditions. This is beneficial for various regions to formulate reasonable urban public health development strategies according to local conditions.

## 2 Research design

### 2.1 Model setting

In order to test the impact of tourism cooperation on urban public health development, this paper constructs the following benchmark regression model:


(1) $$\mathrm{EL}P_{\mathrm{it}}=\eta_{0}+\eta_{1}RES_{\mathrm{it}}+\eta_{2}X_{\mathrm{it}}+\mu_{i}+\lambda_{t}+\varepsilon_{\mathrm{it}}$$

In model (1), *i* represents the city, *t* represents time, and *ELP* represents the level of public health development in the city; *RES* represents the degree of tourism cooperation, *X* represents a series of control variables, and *μ*, *λ*, and *ε* represent regional effects, time effects, and random disturbance terms, respectively.

To test the transmission effect of smooth internal circulation on the impact of tourism cooperation on urban public health development, the following mediation effect model is set up:


(2) $$IC_{\mathrm{it}}=\vartheta_{0}+\vartheta_{1}RES_{\mathrm{it}}+\vartheta_{2}X_{\mathrm{it}}+\mu_{i}+\lambda_{t}+\varepsilon_{\mathrm{it}}$$

(3) $$\mathrm{EL}P_{\mathrm{it}}=\nu_{0}+\nu_{1}RES_{\mathrm{it}}+\nu_{2}IC_{\mathrm{it}}+\nu_{3}X_{\mathrm{it}}+\mu_{i}+\lambda_{t}+\varepsilon_{\mathrm{it}}$$

Among them, *IC* represents the index of smooth internal circulation. If *RES* in model (2) has a significant positive effect on *IC*, and the coefficient of *RES* on *ELP* in model (3) is not significant or has decreased compared to model (1), it indicates that smooth internal circulation is an important channel for tourism cooperation to empower urban public health development.

### 2.2 Variable definition and explanation

Core explanatory variable: tourism cooperation degree. The measurement of the degree of tourism cooperation is an important topic in tourism research, involving multiple aspects such as destination management, regional cooperation, and stakeholder coordination. Tourism cooperation usually refers to the collaboration among different stakeholders (such as government, enterprises, communities, etc.) to achieve common goals. This article believes that tourism cooperation has a strong systemic connotation, which is essentially a process of coupling different regional tourism subsystems to form higher-level structures and functions. The degree of tourism cooperation is characterized by the value-added part of the operational efficiency of each regional tourism subsystem integrated into a regional large-scale system based on collaborative effects. The formula is as follows:


(4) $$\left\{ \begin{array}{c} RES_{ij}=\left[\left(\theta_{ij}^{\prime }-{\overset{-}{\theta }}_{ij}^{\prime }\right)-\left(\theta_{i}^{\prime }-{\overset{-}{\theta }}_{n}^{\prime }\right)\right]+\left[\left(\theta_{ij}^{\prime }-{\overset{-}{\theta }}_{ij}^{\prime }\right)-\left(\theta_{j}^{\prime }-{\overset{-}{\theta }}_{n}^{\prime }\right)\right]\\ RES_{i}=\overset{n}{\underset{j=1}{\sum }}RES_{ij}\left(j=1,2,\cdots ,n;i\neq j\right) \end{array}\right.$$

Among them, $RES_{ij}$ represents the intensity of tourism synergy between city *i* and city *j*, and $RES_{ij}$ represents the total intensity of synergy between city i and other cities, that is, the degree of tourism cooperation and development; $\theta_{i}^{\prime }$ and $\theta_{j}^{\prime }$ respectively represent the efficiency of independent tourism systems in cities *i* and *j*, while $\theta_{ij}^{\prime }$ represents the overall tourism efficiency of cities *i* and *j*; ${\overset{-}{\theta }}_{ij}^{\prime }$ and ${\overset{-}{\theta }}_{n}^{\prime }$ respectively represent the sample mean of overall tourism efficiency and individual city tourism efficiency. The tourism efficiency values included are calculated by the SBM model and its GML index. The input indicators for measuring tourism efficiency are the number of tourism industry employees and fixed asset inputs, while the output indicator is the total tourism revenue.

Dependent variable: Development level of urban public health. Construct an evaluation index system for the target level of public health in Chinese cities from two dimensions: public health foundation and public health performance, and select 9 measurement indicators for construction. Dietary nutrition level is an indispensable part of evaluating residents’ public health, and the per capita seafood consumption index is selected to characterize residents’ dietary nutrition level. The basic health literacy of residents is measured by their basic education level, specifically expressed by the number of full-time secondary school teachers per 10,000 people. Since the regional infectious disease data is only available at the provincial level, and the incidence rate of infectious diseases among residents is positively related to the urbanization rate, and negatively related to the level of science and technology, this study selects the urbanization rate and the number of patent grants as weights to empower the incidence rate data of infectious diseases in all provinces (regions) in China, so as to calculate the incidence rate data of infectious diseases in all cities in China, and use the projection pursuit method to calculate the level of urban public health development (34, 35).

Control variables: To avoid the problem of omitted variables, this article selects control variables from five levels: economic vitality, fiscal decentralization, opening up to the outside world, financial support, and economic density. Economic Vitality (EV): Expressed as NPP-VIRS Nighttime Light Index. Fiscal decentralization (FD): expressed as the ratio of government general fiscal expenditure to general fiscal revenue. External Development (OP): Expressed as the ratio of actual use of foreign capital to regional GDP. Financial Development (FI): Expressed as the logarithm of the year-end deposit balance of financial institutions. Innovation Capability (CA): Expressed as the ratio of year-end patent grants to the resident population. Economic density (ED): expressed as the ratio of gross domestic product to regional area (37, 38) (Table 1).

**Table 1:** Variable description and descriptive statistics.

Variable type	Variable name	Indicator	*N*	Mean	SD	Min	Max
Explained variable	Tourism cooperation degree	Regional large-scale system based on collaborative effects	3,408	0.1532	0.0812	0.0153	0.5975
Core explanatory variable	Urban public health	Select 9 measurement indicators for construction	3,408	0.4919	0.4999	0.0000	1.0000
Control variables	Economic vitality	Expressed as NPP-VIRS Nighttime Light Index	3,408	0.3871	0.1910	0.0551	0.8975
	Fiscal decentralization	Expressed as the ratio of government general fiscal expenditure to general fiscal revenue	3,408	0.3429	0.2709	0.0000	0.8705
	External development	Expressed as the ratio of actual use of foreign capital to regional GDP	3,408	0.3925	0.2085	0.0000	0.8889
	Financial development	Expressed as the logarithm of the year-end deposit balance of financial institutions	3,408	2.8638	0.3649	0.0000	4.1897
	Innovation capability	Expressed as the ratio of year-end patent grants to the resident population	3,408	0.4238	0.3126	0.0145	0.9231
	Economic density	Expressed as the ratio of gross domestic product to regional area	3,408	0.2530	0.2605	0.1247	0.6364

### 2.3 Data sources and descriptive statistics

The research object of this article is 284 prefecture level cities in China from 2011 to 2022. The data sources mainly include the EPS database of the corresponding year, the China Urban Statistical Yearbook, the statistical yearbooks of various provinces and cities, the statistical bulletins of various cities, and the Guotai An database. A small amount of missing data is filled in using linear trend method.

## 3 Empirical result analysis

### 3.1 Benchmark regression results

Before conducting empirical analysis, this article uses correlation coefficients and variance inflation factors to test for multicollinearity among variables. The results showed that the correlation coefficients between variables were all below 0.7, and the VIF values were all below 2, far less than 10, indicating that the regression model in this article does not have serious multicollinearity problems.

Columns (1)–(6) of Table 2 show the benchmark regression results of tourism cooperation on urban public health development after gradually adding control variables. The results showed that in the process of adding control variables one by one, the regression coefficient of tourism cooperation on urban public health development was significantly positive at least at the 5% level, indicating that tourism cooperation can effectively promote urban public health development. This is because in recent years, the Chinese government has deployed major regional strategies, such as the coordinated development of Beijing Tianjin Hebei, the development of the Yangtze River Economic Belt, the construction of the Guangdong Hong Kong Macao Greater Bay Area, and the integrated development of the Yangtze River Delta. It has also continuously improved the platform, system, and legislative guarantees for tourism cooperation. As a result, production factors have been able to flow in an orderly manner, the mechanism for distributing benefits has become increasingly reasonable, and the regional economic layout and territorial spatial system with complementary advantages have gradually taken shape, leading to a rapid increase in tourism cooperation. The improvement of tourism cooperation can promote the construction of a modern industrial system by strengthening economic connections and industrial division of labor, thereby providing an industrial foundation for the development of urban public health. Secondly, tourism cooperation usually requires cities to improve and enhance their tourism environment, including urban greening, beautification, purification, and other aspects. These measures help improve the urban environment and enhance the quality of life for residents and tourists. A good urban environment helps to reduce the occurrence and spread of diseases, thereby improving the public health level of the city. The third is to effectively integrate resources and fully tap into comparative advantages to promote the emergence of disruptive technological innovation, thereby providing a technological foundation for the development of urban public health. The fourth is to rely on the scale borrowing effect and factor flow effect between regions to promote the virtual agglomeration of new production factors, thereby providing a factor foundation for the development of urban public health, and thus fully empowering the development of urban public health.

**Table 2:** Benchmark regression results.

	(1)	(2)	(3)	(4)	(5)	(6)
*RES*	0.022*** (2.57)	0.015** (2.12)	0.016** (2.44)	0.054* (2.45)	0.043* (2.22)	0.016** (2.22)
*EV*		0.023*** (21.20)	0.033** (20.21)	0.027*** (19.45)	0.017*** (11.32)	0.012*** (11.44)
*FD*			−0.003*** (−3.45)	−0.002*** (−3.89)	−0.002*** (−3.43)	−0.002*** (−3.56)
*FI*				0.126** (2.33)	0.010** (2.17)	0.010** (2.19)
*CA*					0.116** (31.77)	0.128*** (39.14)
*ED*						0.834*(1.77)
*Cons*	0.274*** (1,338)	0.177*** (68.10)	0.182*** (53.59)	−0.025 (−0.27)	0.018 (0.27)	0.164 (0.25)
Individual fixed effects	Fixed	Fixed	Fixed	Fixed	Fixed	Fixed
Fixed time effect	Fixed	Fixed	Fixed	Fixed	Fixed	Fixed
*R* ^2^	0.110	0.242	0.256	0.267	0.482	0.476
Observations	3,408	3,408	3,408	3,408	3,408	3,408

*, **, *** are divided into significance levels of 1, 5, and 10%.

In terms of controlling variables, the impact of economic vitality, financial development, innovation capability, and economic density on the development of urban public health is significantly positive, indicating that the four have a promoting effect on the development of urban public health. This is because economic vitality can promote the aggregation of high-quality labor force, thereby reducing the production factor constraints on urban public health development. Financial development can reduce the financing constraints on the future development of industries and strategic emerging industries, as well as the transformation of traditional industries, thereby accelerating the construction of a modern industrial system and promoting the development of urban public health. Innovation capability can promote the emergence of disruptive technological innovation to support the development of urban public health. Economic density can form economies of scale and labor pool effects to support the upgrading of industrial structure, which in turn will promote the construction of a modern industrial system and empower the development of urban public health. The impact of fiscal decentralization on urban public health development is significantly negative, mainly because fiscal decentralization may lead to vicious competition among local governments in order to attract investment and promote economic growth, thereby reducing resource allocation efficiency and inhibiting urban public health development.

### 3.2 Endogeneity and robustness test

#### 3.2.1 Endogenous test

The higher the level of urban public health development, the better the innovative allocation of production factors and the deep transformation ability of industries, which is conducive to tourism cooperation. Therefore, this article uses multiple instrumental variables to alleviate endogeneity issues. Firstly, using Confucian cultural intensity as an instrumental variable, this instrumental variable can influence the tourism cooperation degree of the city through the shaping effect of social and cultural atmosphere, satisfying correlation, but not directly related to the urban public health development status of the city, satisfying exclusivity. The regression results are shown in column (2) of Table 3. Secondly, the mean of regional synergy among other cities in the same province in the same year is used as the instrumental variable. This instrumental variable can affect the tourism cooperation degree of the city through demonstration and communication effects, satisfying correlation, but not directly related to the urban public health development status of the city, satisfying exclusivity. The regression results are shown in column (3) of Table 2. The results show that in columns (1)–(2) of Table 3, the *p*-values of the Anderson canon. Corr. LM statistic are all 0.000, rejecting the null hypothesis of insufficient identification of instrumental variables. The Cragg Donald Wald F statistic is greater than the critical value of weak instrumental variables at the 10% level, rejecting the null hypothesis of weak instrumental variables. It can be seen that considering endogeneity issues from multiple dimensions, tourism cooperation can still significantly promote the development of urban public health.

**Table 3:** Endogenous test.

	(1)	(2)
*RES*	0.556*** (4.45)	0.338*** (9.21)
Control variable	Control	Control
Individual fixed effects	Fixed	Fixed
Fixed time effect	Fixed	Fixed
Anderson canon. Corr. LM	24.562 [0.000]	75.68 [0.000]
Cragg-Donald Wald F	32.463 {16.54}	78.459 {16.54}
*R* ^2^	0.572	0.498
Observations	3,408	3,408

The *t*-value calculated by the urban clustering standard error is included, and *** is at a significance level of 1%. [] represents the *p*-value, {} represents the 10% critical value for weak instrumental variable testing.

#### 3.2.2 Robustness test

To further verify the robustness of the conclusions in this article, the following method is used for robustness testing: (1) replacing the dependent variable. The comprehensive indicator system is constructed from three dimensions of population survival rate, urban infectious disease incidence rate and life expectancy, and the development level of urban public health is measured by entropy weight method, and then regressed again. The results are shown in column (1) of Table 4. (2) Replace explanatory variables. Replace the SBM model used to measure the operational efficiency of regional economic systems in the efficiency appreciation model with the EBM model, recalculate the degree of tourism cooperation, and regress. The results are shown in column (2) of Table 4. (3) Tail truncation processing. To alleviate the impact of extreme values on the estimation results, this study performed a 1% bilateral truncation on the research sample and re regressed. The results are shown in column (3) of Table 4. (4) Adjust the sample period. To avoid the impact of the epidemic, the research period was shortened from 2011 to 2022 and the regression was conducted again after 2011 to 2019. The results are shown in column (4) of Table 4. (5) Exclude special samples. Considering that the administrative level of municipalities directly under the central government may cause disturbance to the results, Beijing, Tianjin, Shanghai, and Chongqing were excluded from the research sample and the regression was conducted again. The results are shown in column (5) of Table 4. After conducting a series of robustness tests, it was found that tourism cooperation still has a significant promoting effect on the development of urban public health, which is basically consistent with the benchmark regression results, indicating that the conclusion of this article is robust.

**Table 4:** Robustness test.

	(1)	(2)	(3)	(4)	(5)
*RES*	0.074** (2.34)	0.127*** (6.38)	0.011*** (3.15)	0.014* (1.89)	0.013** (2.61)
*Cons*	0.164 (0.21)	4.287*** (6.28)	0.132*** (2.72)	0.077 (1.01)	0.067* (1.95)
Control variable	Control	Control	Control	Control	Control
Individual fixed effects	Fixed	Fixed	Fixed	Fixed	Fixed
Fixed time effect	Fixed	Fixed	Fixed	Fixed	Fixed
*R* ^2^	0.432	0.377	0.382	0.337	0.423
Observations	3,408	3,408	3,408	2,556	3,360

*, **, *** are divided into significance levels of 1, 5, and 10%.

#### 3.2.3 Heterogeneity analysis

The differences in cooperation modes may lead to heterogeneity in the urban public health empowerment effects generated by tourism cooperation. This article focuses on examining the differential empowerment effects of two types of cooperation models on urban public health development: one is the adjacent city type cooperation model, where cities establish cooperative relationships with neighboring cities adjacent to their administrative regions to achieve tourism cooperation. The second is the “core hinterland” cooperation model, in which the provincial capital cities and deputy provincial capital cities within the province play a radiating and driving role, while other hinterland cities within the province establish cooperative relationships with these core cities to achieve tourism cooperation. On the basis of Equation (4), different types of spatial adjacency matrices were introduced to obtain the tourism cooperation degree of each city under two types of modes, and heterogeneity tests were conducted. The results are shown in columns (1)–(2) of Table 5. The results indicate that tourism cooperation has a significant positive impact on the development of urban public health in different cooperation models. However, compared to the adjacent city cooperation model, the tourism cooperation formed by the “core hinterland” cooperation model has a greater empowering effect on the development of urban public health. Possible reasons are as follows: On the one hand, although the communication and transaction costs of establishing tourism cooperation between cities and surrounding areas are lower, there may be a phenomenon of homogenization of tourism resources, which masks the empowering effect of tourism cooperation on urban public health development. On the other hand, core cities such as provincial capitals and vice provincial capitals usually have more developed infrastructure, more complete tourism industry chains, and richer tourism resources, which means they have a first mover advantage in the development of urban public health. The promotion of the “core hinterland” cooperation model is conducive to guiding the division of tourism and industry between core cities and hinterland cities, improving the efficiency of tourism resource allocation in core cities, forming a highland for urban public health development, and ultimately relying on channels such as industrial chains, logistics chains, and division of labor networks to spread relevant achievements in core cities that are conducive to urban public health development to hinterland cities, driving the upgrading of tourism in hinterland cities, and ultimately promoting the overall level of urban public health development in the region.

**Table 5:** Heterogeneity test.

Variables	Differences in cooperation modes		Differences in cooperation entities		
	(1)	(2)	(3)	(4)	(5)
*RES*	0.005** (2.16)	0.026** (4.16)	0.033** (3.05)	0.043** (4.54)	0.003** (2.43)
*Cons*	0.018 (0.18)	0.023*** (3.23)	0.026*** (5.22)	0.025 (1.87)	0.064* (2.32)
Control variable	Control	Control	Control	Control	Control
Individual fixed effects	Fixed	Fixed	Fixed	Fixed	Fixed
Fixed time effect	Fixed	Fixed	Fixed	Fixed	Fixed
*R^2^*	0.452	0.552	0.225	0.352	0.252
Observations	3,408	3,408	420	2,988	2,988

*, **, *** are divided into significance levels of 1, 5, and 10%.

The cooperative effects generated by the interactions between different types of urban economic subsystems may vary, leading to further heterogeneity in the empowering effects of urban public health. Therefore, based on the differences in cooperation entities, this article divides them into economic cooperation between central cities, tourism cooperation between central cities and non-central cities, and economic tourism cooperation between non central cities and non central cities. On the basis of adjusting the selection rules of cooperation objects for each city’s tourism subsystem, the heterogeneity test is conducted to obtain the degree of tourism cooperation between different types of cities. The results are shown in Table 5 (3)–(5). The results indicate that tourism cooperation among different entities has a significant positive impact on the development of urban public health. However, the urban public health development empowerment effect of tourism cooperation between central cities is the greatest, followed by central cities and non central cities, and the smallest between non central cities and non central cities. Possible reasons are as follows: Central cities usually have richer human capital, technological resources, and information flow. Therefore, when central cities cooperate with each other in tourism, the sharing power and complementarity of tourism are stronger, which can strengthen the innovation and industrial upgrading effects of tourism cooperation, thereby promoting the formation of larger urban public health. There is a gap between non central cities and central cities in terms of tourism, tourism scale, and tourism support. However, in the process of tourism cooperation with central cities, we can rely on the spillover effects of central cities and the sharing effects of tourism market scale to make up for the shortcomings of non-central cities in terms of tourism resources scarcity and insufficient scale, thereby promoting the overall level of urban public health development in the region. In addition, in the early stages of urban public health development, non-central cities may also face the problem of forced siphoning of urban public health development resources. Therefore, the urban public health empowerment effect generated by tourism cooperation between non central cities and non-central cities is the lowest.

## 4 Conclusions and policy recommendations

Can tourism cooperation empower the development of urban public health? To answer this question, this article takes 284 cities in China from 2011 to 2022 as the research object, empirically explores the impact of tourism cooperation on urban public health development and the mediating role of smooth internal circulation in it. Research has found that tourism cooperation can empower the development of urban public health. After a series of robustness tests, this conclusion still holds true. Secondly, heterogeneity analysis shows that compared to the adjacent city type collaborative model, the tourism cooperation formed by the “core hinterland” collaborative model has a greater empowering effect on the development of urban public health; The synergy between central cities and central city tourism has the greatest empowering effect on urban public health development, followed by central cities and non-central cities, with non-central cities and non-central cities having the smallest. Based on the above conclusions, this article proposes the following policy recommendations:

Firstly, promote the balanced development of tourism and public health. One is to strengthen public health management and services. Establish a sound public health management system, strengthen disease monitoring, early warning, and prevention and control work. Enhance medical treatment capabilities, improve emergency systems, and ensure that tourists and residents receive timely and effective treatment in the event of public health emergencies. The second is to optimize the tourism environment. Strengthen urban greening, beautification, and purification work to improve the quality of the tourism environment (39). Strengthen the supervision of tourist attractions to ensure a clean, safe, and orderly environment. The third is to promote the development of the health tourism industry. Encourage and support the development of the health tourism industry, such as special projects such as hot spring therapy and forest health preservation. Strengthen the planning and management of the health tourism industry to ensure its sustainable development. The fourth is to balance resource allocation. Balancing the development of the tourism industry with other public service sectors in tourism cooperation, ensuring the rational allocation and utilization of resources. Increase investment in public health, education, and other fields to improve the overall level of public services in cities.

Secondly, deepen tourism cooperation and eliminate the regional economic differentiation dilemma of urban public health development. This study indicates that tourism cooperation can effectively enhance the level of urban public health development. Therefore, one suggestion is to accelerate the improvement of the division of labor system in the tourism industry among cities, promote infrastructure interconnection, facilitate the sharing of public services such as education and healthcare, and further enhance the level of tourism cooperation. The second is to encourage the use of the digital economy to empower the tourism market as a lever, collaborate to build a first-class business environment and improve the digital tourism market system and mechanism, accelerate the construction of a cross regional, multi-level, and multi-dimensional integrated tourism market system, and further strengthen the radiation path of tourism cooperation and development (40). The third is to accelerate the construction of a high-quality regional integration institutional system, strengthen the coordination and cooperation between regional tourism policies and policies in finance, currency, industry, and other aspects, and further promote the level transition of positive externalities in tourism cooperation.

Thirdly, optimize the collaborative development strategy and improve external guarantee conditions simultaneously, fully developing the urban public health development empowerment role of tourism cooperation (41). Firstly, this article finds that in the “core hinterland” collaborative model, tourism cooperation has a greater empowering effect on the development of urban public health. Therefore, on the one hand, it encourages the deep integration of the tourism industry chain upstream and downstream between core cities and hinterland cities, and further builds a tourism division of labor network based on the tourism industry chain; On the other hand, it is recommended that cities with similar advantages in tourism resources establish a tourism resource conversion community and develop a unified market competition mechanism to promote the free flow of factors such as labor, capital, land, and data, thereby alleviating the masking effect of homogeneous competition on the positive externalities of tourism cooperation. Secondly, this article found that different types of urban tourism synergy have different empowering effects on the development of urban public health. Therefore, on the one hand, it is necessary to encourage central cities to formulate more flexible fiscal and tax policies and strengthen their leading role in the development of urban public health; On the other hand, it is necessary to support the development of characteristic tourism economy and industries in non central cities, while strengthening the tourism connection with central cities, and promoting the characteristic and coordinated development of public health in non-central cities.

## Data Availability

Publicly available datasets were analyzed in this study. This data can be found here: https://www.shujuku.org.
